# Use of heat and moisture exchanger in intubated patients reduces the blockage in gas sampling tube of the mass spectrometer

**DOI:** 10.4103/1658-354X.76469

**Published:** 2011

**Authors:** Nataraj Madagondapalli Srinivasan

**Affiliations:** *Department of Anesthesiology, Kasturba Medical College, Manipal, India*

Sir,

The use of mass spectrometers for gas monitoring during anesthesia has become a standard anesthetic practice. The attractiveness of mass spectrometer technology comes from its fundamental capability of measuring a large number of gas constituents simultaneously with precision and high speed. The sampling tube which delivers the gases from the patient end can get blocked by the condensed water or secretions in endotracheal tube. We describe our observation to circumvent the problem.

The mass spectrometer works by continuously suctioning the gases from the patient end via a gas sampling tube connected to the luer lock port of the “L” connector between the endotracheal tube and breathing circuit. The gas sampling tube from the patient to the mass spectrometer can be blocked because of secretions or defects which can cause the spectrometer to malfunction.[1]

It is observed that during general anesthesia, the gas sampling tube frequently gets blocked with the condensed water droplets or secretions present in the endotracheal tube.[1] Even though the manufacturer provides water trap at the point of entry of gases into mass spectrometer through D-Fend^®^, the monitor gives an alarm as “blocked sample line – check D-Fend” and it stops analyzing any further or gives erroneous reading. Also, the capnograph trace is disrupted. This hinders the monitoring and impairs decision making in a critical event.

There are several ways of mitigating the problem. The simplest defense is to use high (dry) fresh gas flows. But this may not be feasible when low flow anesthetic techniques are being employed. Another approach is to use a short length of tubing designed by E for M Corp, Germany (being bought by Marquette Electronics- Milwaukee, Wisconsin 53223, U.S.A.) as a Patient Sample Dryer. It actively exchanges water with the air outside the sample line. The short length of tubing may not be ideal for surgeries which require anesthesia work station positioned at a far end. We have observed that connecting a heat moisture exchanger (HME) between the endotracheal tube and the gas sampling tube prevents the blockage of the sampling line and the D-Fend. The HME absorbs the minute water droplets entering the sampling port and sampling tube; in addition, the HME also provides heat and moisture exchange for inspired gas and acts as bacterial filter.[2]

Based on the above observation, we recommend routine use of the HME for all patients undergoing general anesthesia, irrespective of the duration of surgery. Even though they add to the cost for the patient, we feel it is worthwhile using HME as the uninterrupted data provided by mass spectrometer surpass the cost of the HME.

## References

[1] Skeehan TM, Biebuyck JF (1991). Erroneous mass spectrometer data caused by a faulty patient sampling tube: case report and laboratory study. J Clin Monit.

[2] Rathgeber J (2006). Devices used to humidify respired gases. Respir Care Clin N Am.

